# Supplementary material for: Brightness illusions drive a neuronal response in the primary visual cortex under top-down modulation
Source: Nat Commun. 2024 Apr 23;15:3141. doi: 10.1038/s41467-024-46885-6 (PMC11039481; doi:10.1038/s41467-024-46885-6)
Supplement: Supplementary file 1 — Supplementary Information [file 41467_2024_46885_MOESM1_ESM.pdf]

Supplementary information

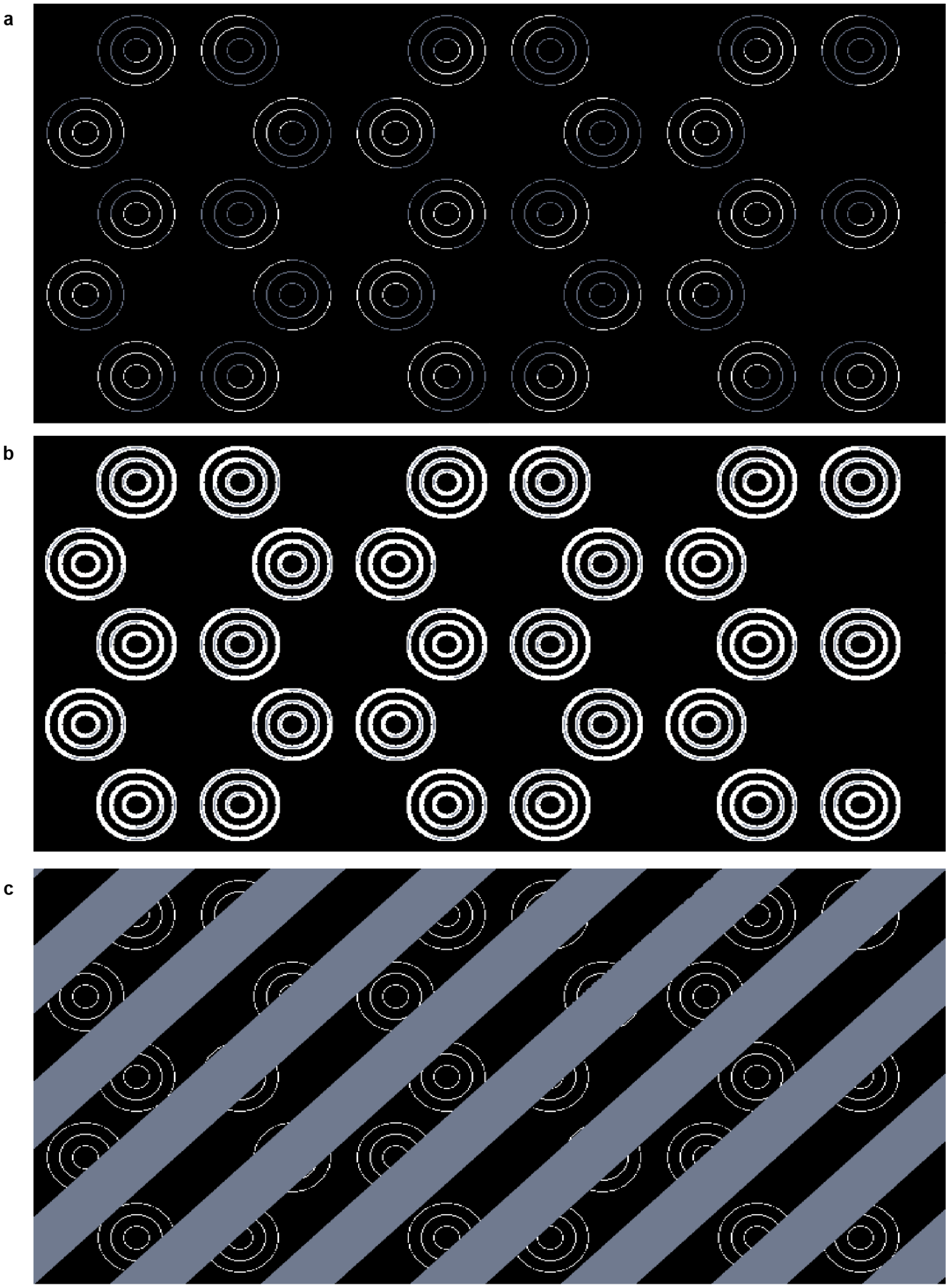

Supplementary Fig. 1. Full-screen version of stimuli used in the main experiment session.
a-c, show the NCS, DBC, and LDG stimuli, respectively.

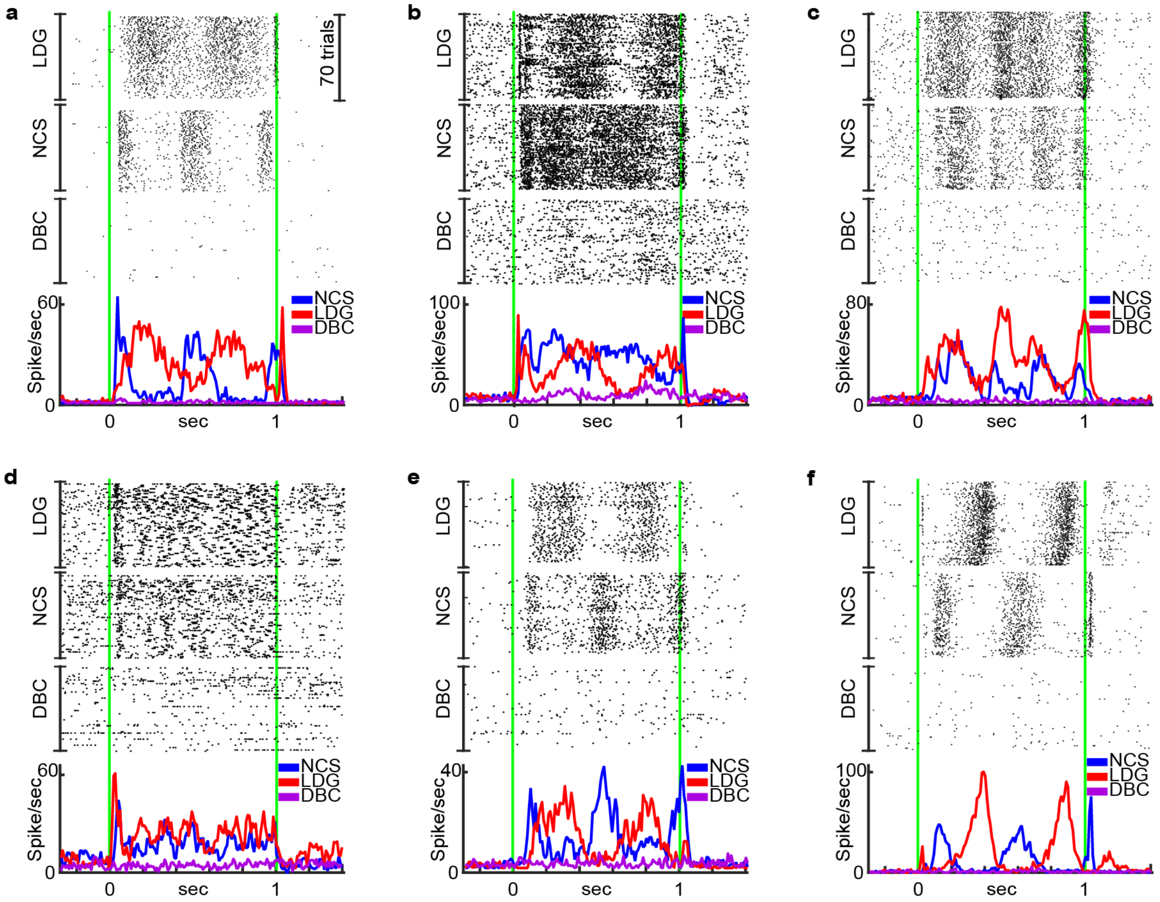

Supplementary Fig. 2. Responses of example single units to different stimuli.
a-f, Rasters, and peri-stimulus time histograms (baseline-subtracted) of V1 single units in response to different stimulus types. The plots show the response to physical gratings (LDG stimuli), illusory gratings (NCS stimuli), and diffusion-blocked illusory gratings (DBC stimuli) presented in the preferred direction of each unit. The green lines indicate the times of stimulus onset and offset.

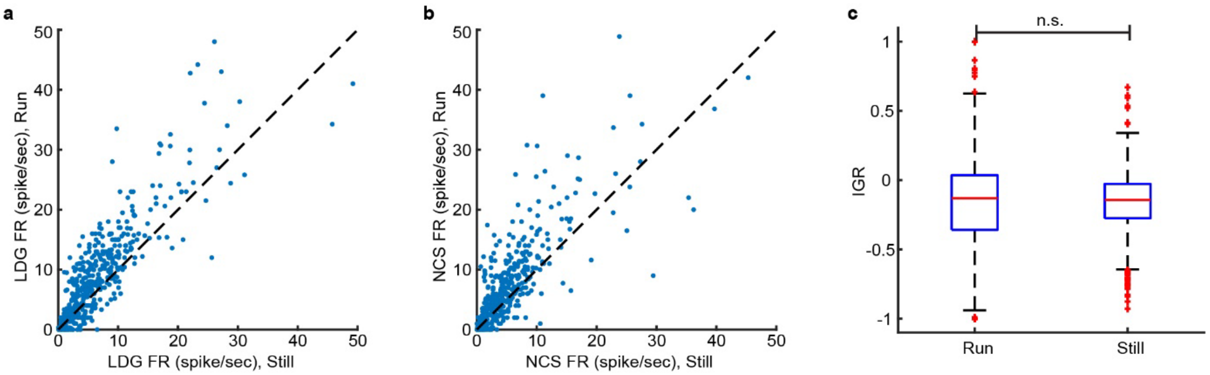

Supplementary Fig. 3. Running does not affect the illusory grating response.
a and b, Scatter plots show the firing rate of V1 neurons in response to LDG and NCS stimulus for "run" and "still" trials. The dashed line represents the identity line. **c,** Box plot shows the IGR index for two run and still trials groups (LMEM: F=2.26, p=0.13). In each box plot, the median is represented by the central mark, and the 25th and 75th percentiles are indicated by the lower and upper edges of the box, respectively. The whiskers extend to the farthest data points that are not classified as outliers, and outliers are individually marked using the '+' symbol. N = 6 mice

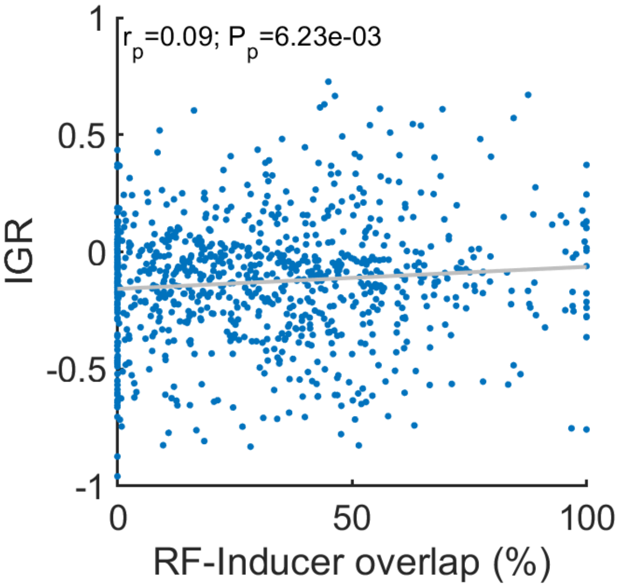

Supplementary Fig. 4.
The scatter plot shows the IGR response against the overlap ratio of the receptive field and the circular patch of the inducer. Each dot presents one single unit. Pearson correlation and its p-value (two-sided t-test) are shown in the figure. The gray line represents a least square fit to the data. N=13 mice.

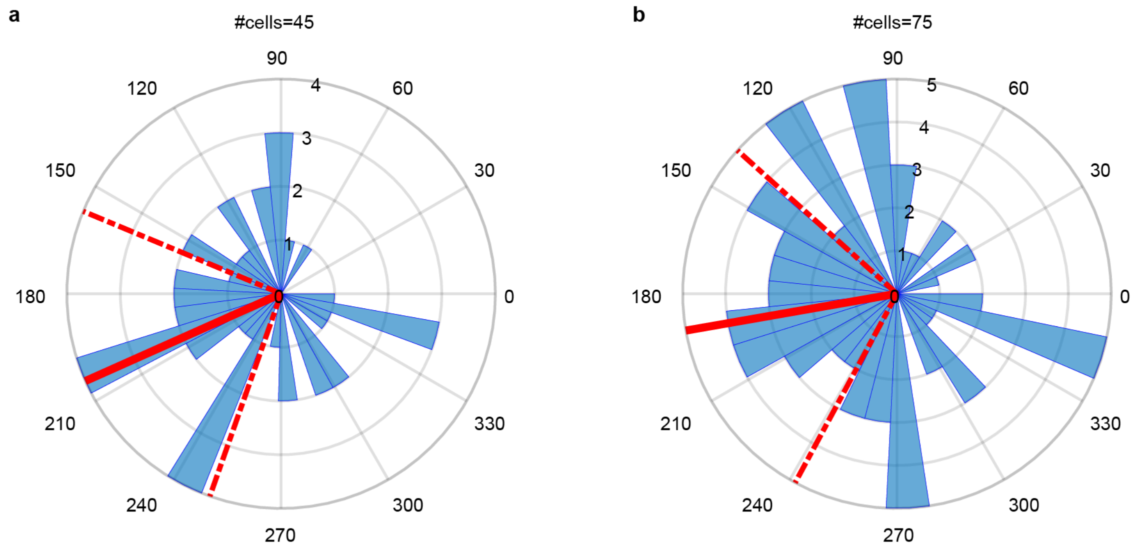

Supplementary Fig. 5. Phase shift analysis for different populations.
a and b, The polar plots show the phase shift between the response evoked by NCS and LDG stimuli for a subset of neurons with RF/inducer overlap of 0.1% and 5% of RF size, respectively. The solid red lines show the angular mean of the circular distributions, and the dashed red lines show a 95% confidence interval of the mean. The radial numbers indicate the number of neurons in the histogram. N=13 mice

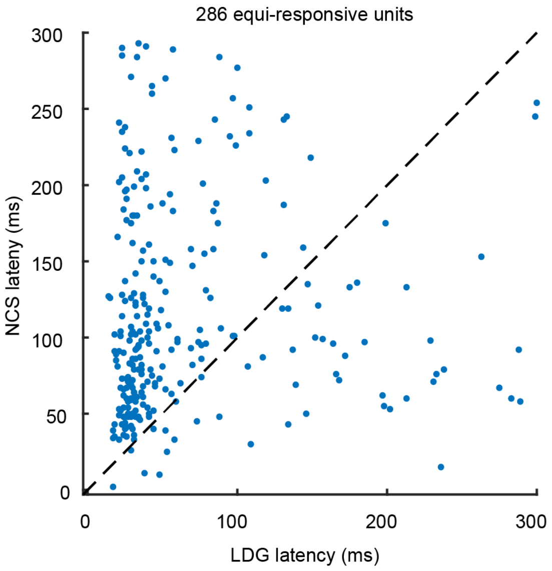

Supplementary Fig. 6. Equi-responsive cells are delayed in response to illusory brightness. The scatter plot shows the response latency of 286 equi-responsive cells to NCS stimulus against LDG stimulus. NCS responses are delayed for equi-responsive unit (LMEM: F=30.74, p=4.31e-8). Equi-responsive cells are the cells with no significant differences in the intensity of their responses to NCS and LDG stimuli. N = 13 mice.

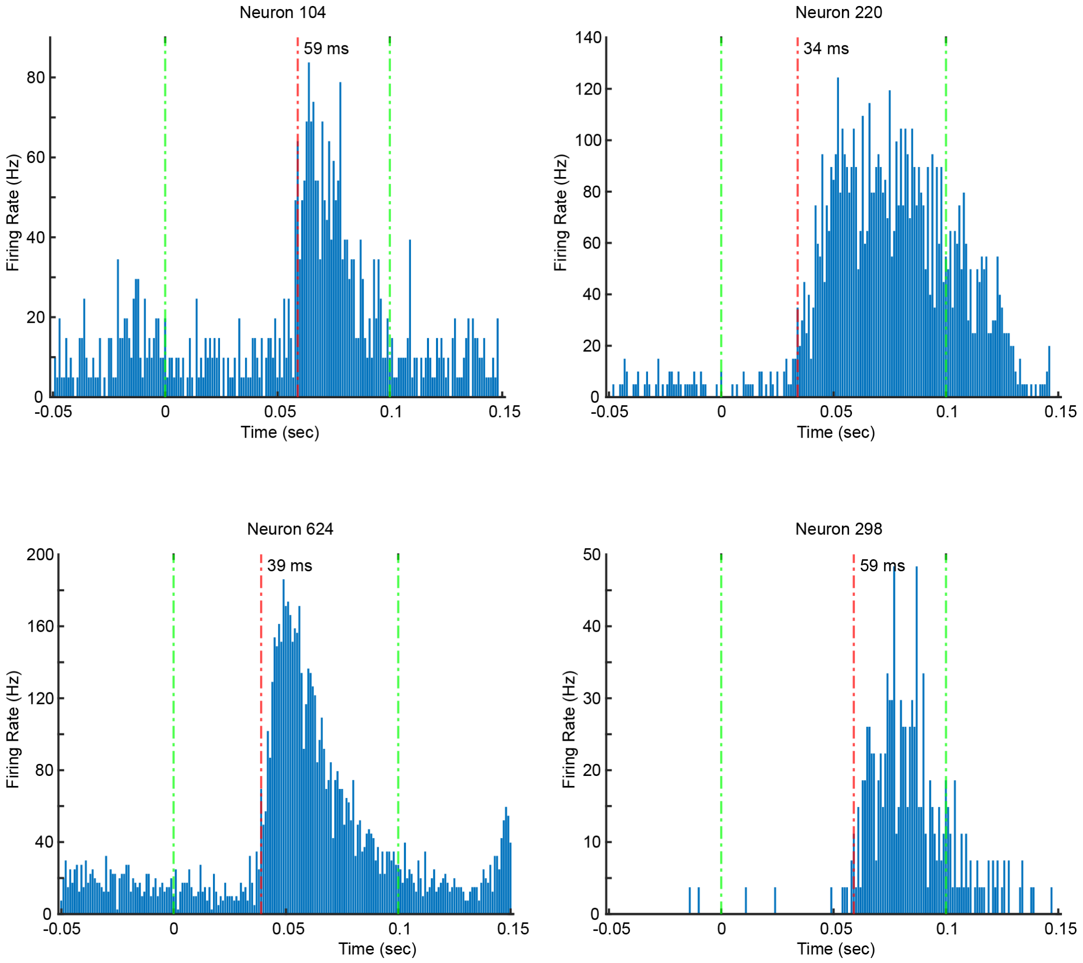

Supplementary Fig. 7. PSTH of some example units and their rectangle latencies
Histograms show examples of single units PSTH in response to flashing rectangles with contrast increment during the RF mapping session. Green Dashed lines show the offset and onset of the stimulus. The red dashed line shows the estimated response latency.

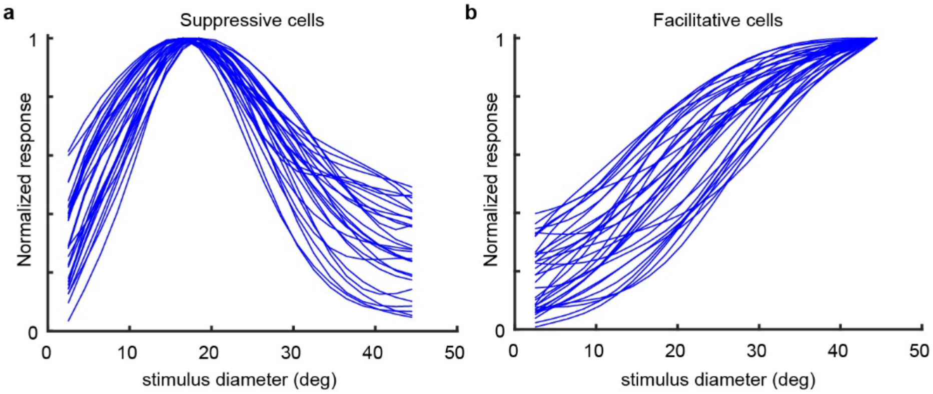

Supplementary Fig. 8. Two types of surround modulation
a, size tuning curve of 30 examples of suppressive cells. **b,** size tuning curve of 30 example facilitative cells. N= 6 mice

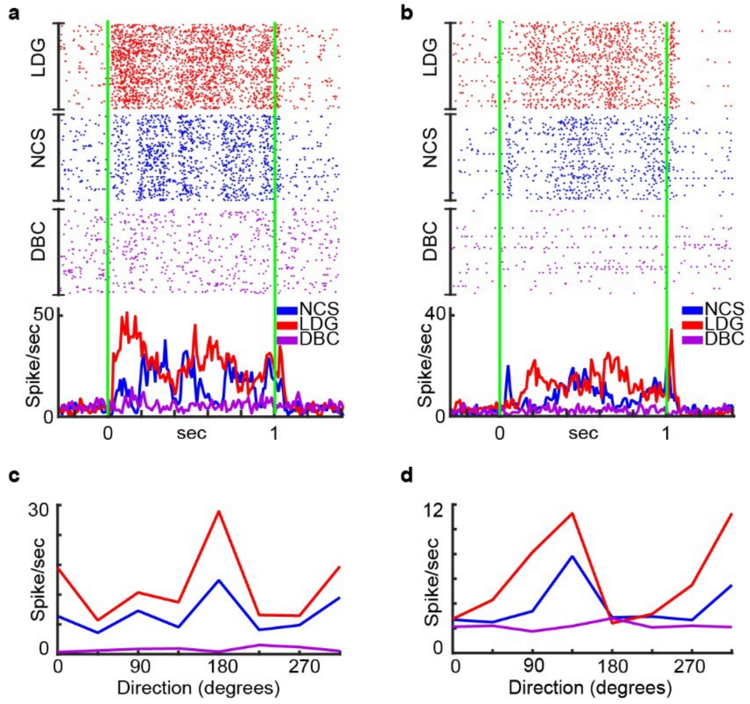

Supplementary Fig. 9. **Example of complex cell responses**

a and b, Raster and peri-stimulus time histogram (baseline-subtracted) of two V1 complex cells (CSM in a: -0.7 and b:-0.6) in response to different stimulus types. The plots show the response to physical gratings (LDG stimuli), illusory gratings (NCS stimuli), and diffusion-blocked illusory gratings (DBC stimuli) presented at the preferred direction of each unit (180° and 135°). Green lines show the stimulus onset and offset. **c and d,** direction tuning curves of the two example neurons in a and b.

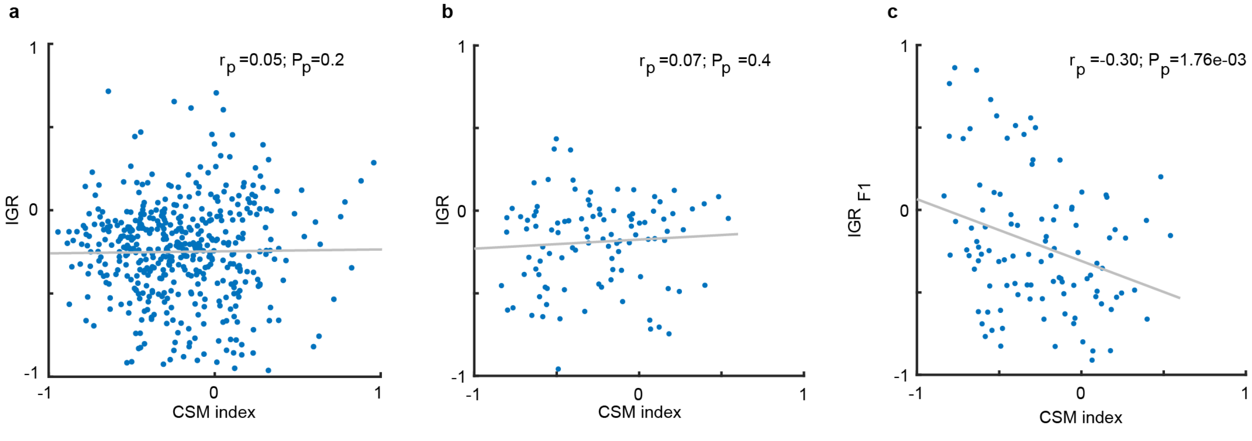

Supplementary Fig. 10. Complex cells contribute more to the NCS processing

a, IGR plotted against complex simple modulation (CMS) index. Each dot presents one single unit. r_p and p_p show Pearson's correlation coefficient and p-value (two-sided t-test), respectively. N= 6 mice. **b,** IGR plotted against CMS index for non-overlapping units. Plot conventions are

the same as in a. N=13 mice. **c,** IGR_{F1} plotted against CMS index for non-overlapping units. Plot conventions are the same as in a. N=13 mice.

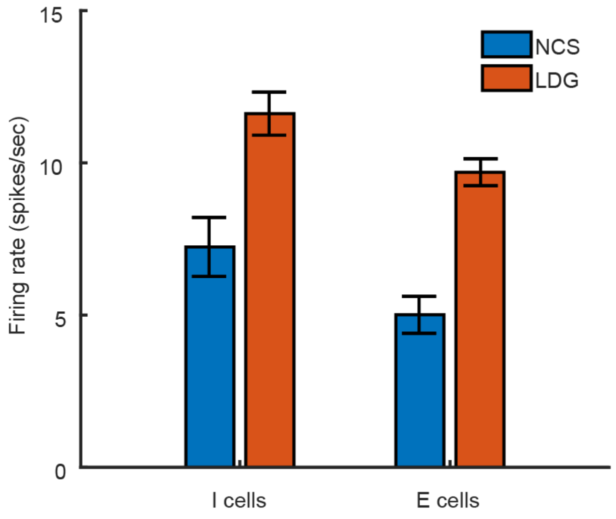

Supplementary Fig. 11.
The bar plot shows the average response of putative inhibitory (n= 146) and excitatory neurons (n= 374) to NCS and LDG stimuli. Error bars show the standard error of the mean values. N= 6 mice.

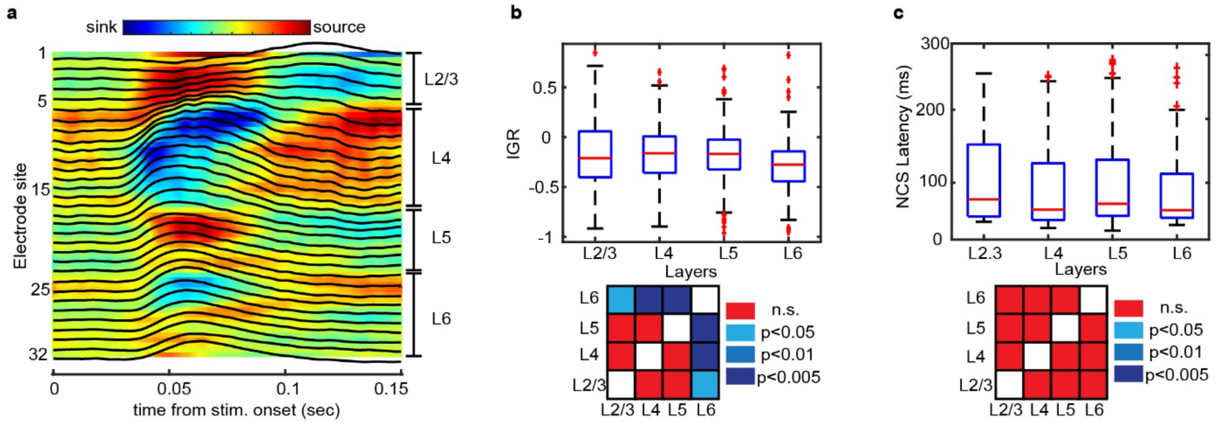

Supplementary Fig. 12. Layer-based analysis of V1 activities.
a, Average current source density (CSD) map and local field potential (LFP) traces for visual stimulation. CSD demonstrates the segregation of responses by layer. Blue represents current sinks, and red represents current sources. Black traces represent LFP. **b,** Box plot (top) shows the neuronal illusory grating responses (IGR) in different layers. The color-coded matrix (bottom) presents the post hoc multiple comparison test results. **c,** Box plot (top) shows the neuronal response latency to NCS illusion in different cortical layers. The post hoc multiple comparisons (bottom) show no layer-specific differences in the NCS response latencies. In each box plot, the median is represented by the central mark, and the 25th and 75th percentiles are indicated by the

lower and upper edges of the box, respectively. The whiskers extend to the farthest data points that are not classified as outliers, and outliers are individually marked using the '+' symbol. N = 6 mice

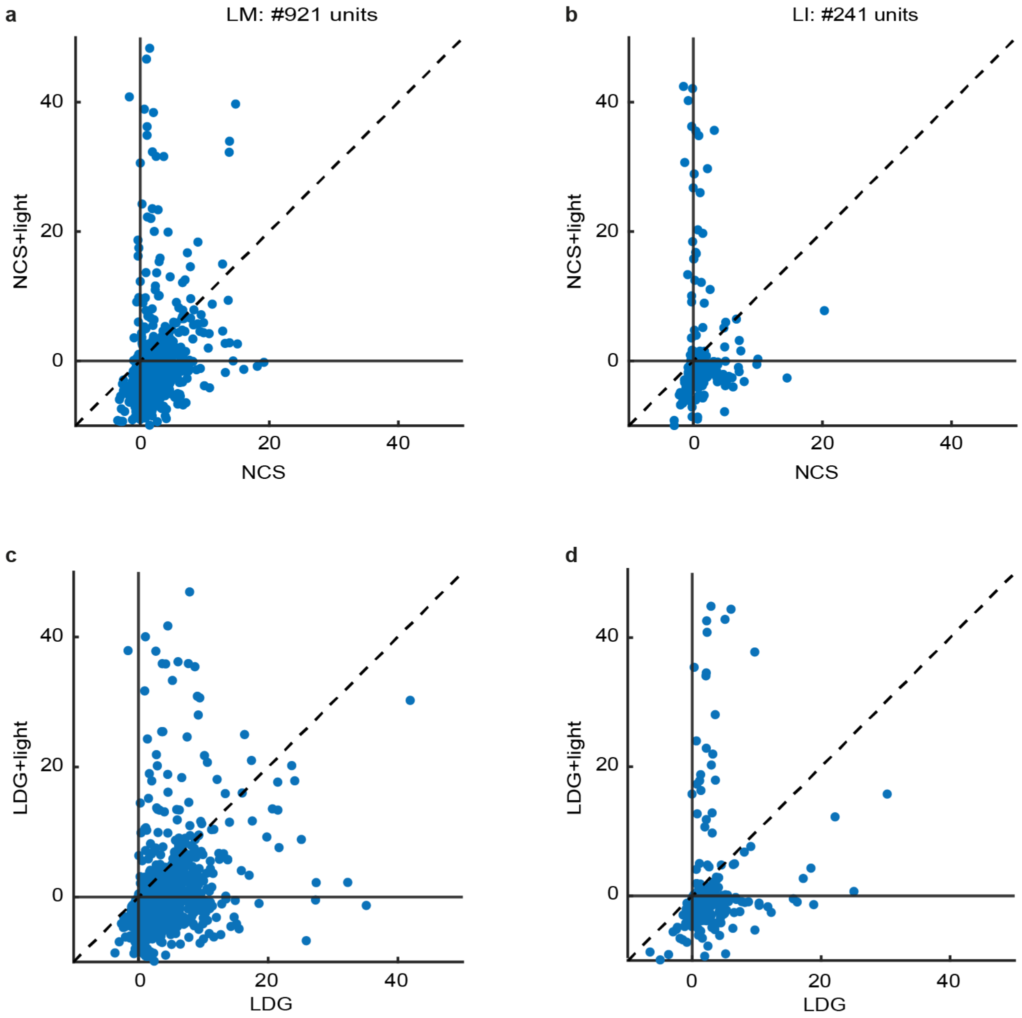

Supplementary Fig. 13. Responses of HVAs neurons to stimuli in optogenetics experiment. **a,** NCS responses plotted against NCS+light responses in LM. Each dot presents the pre-stimulus subtracted firing rates of one single unit (n=921 units). The highly firing unit in the presence of light are likely to be PV+ interneurons. The dashed line represents the identity line. **b,** NCS responses plotted against NCS+light responses in LI (n= 241 units). The plot conventions are the same as in a. **c,** LDG responses plotted against LDG+light responses in LM (n=921 units). The plot conventions are the same as in a. **d,** LDG responses plotted against LDG+light responses in LI (n= 241 units). The plot conventions are the same as in a. N=6 mice.

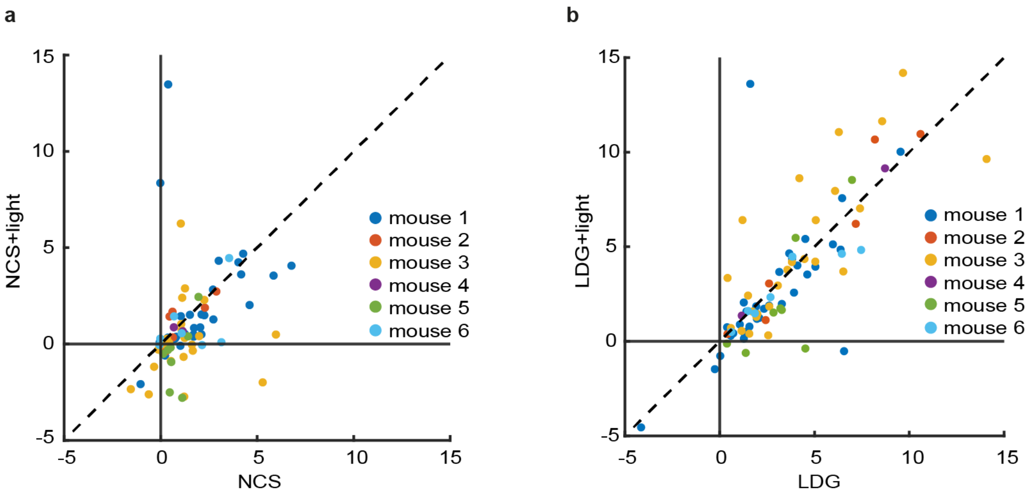

Supplementary Fig. 14. Responses of HVAs neurons to stimuli in optogenetics experiment.

a, NCS responses plotted against NCS+light responses in V1. Each dot presents the pre-stimulus subtracted firing rates of one single unit (n=85 units). The dashed line represents the identity line. **b,** LDG responses plotted against LDG+light responses in V1 (n=85 units). The plot conventions are the same as in a. N=6 mice.

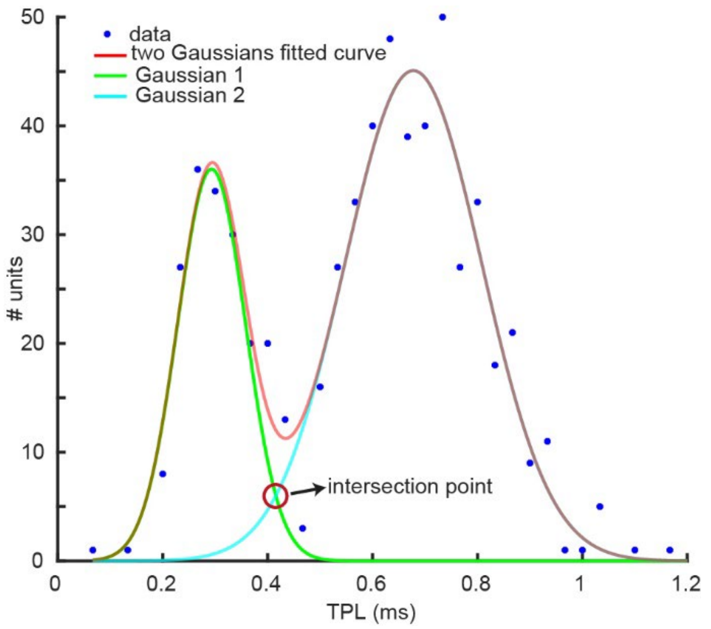

Supplementary Fig. 15. **Classification of putative I/E cells**.
Two Gaussian functions are fitted to the distribution of trough-to-peak latency (TPL), and the intersection point is selected as a threshold. Cells with a TPL shorter than the threshold are considered as narrow-waveform inhibitory cells. Other cells are regarded as wide-waveform principal cells (i.e. putative excitatory cells).

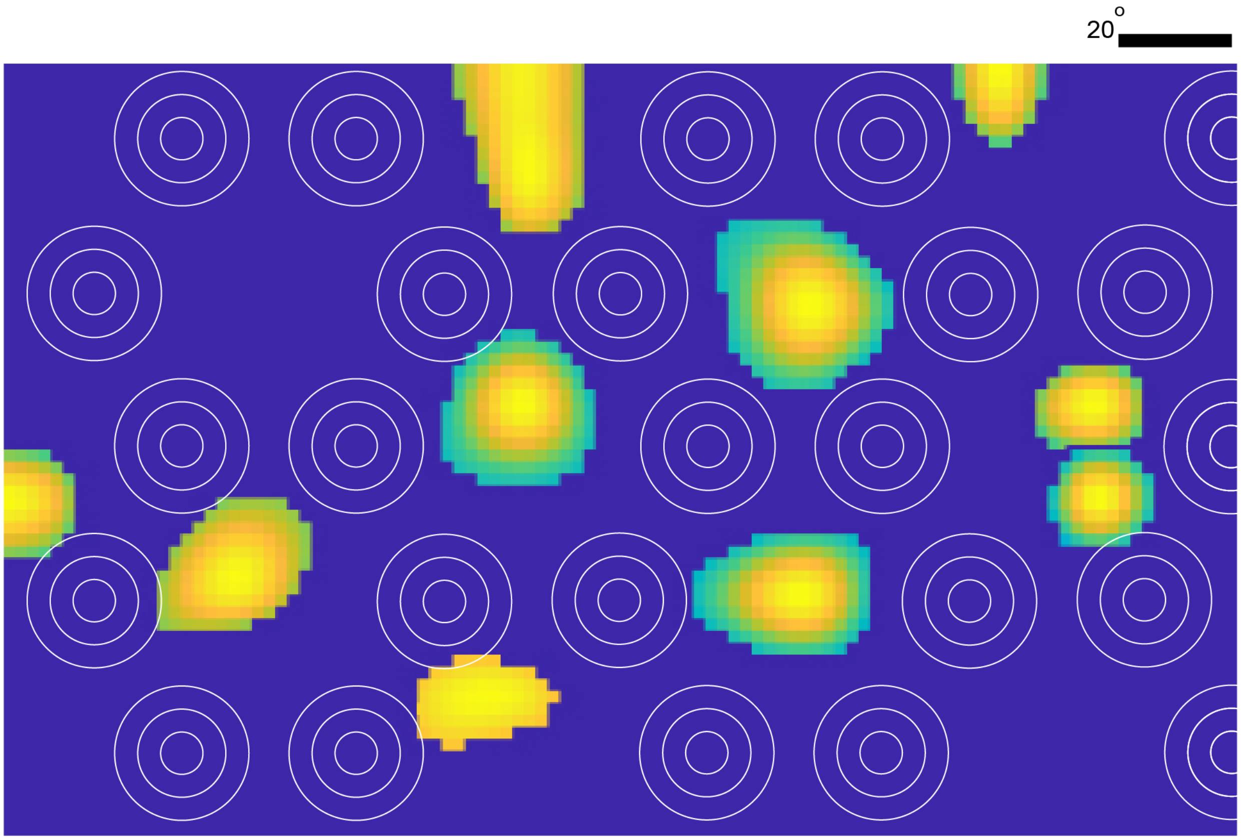

Supplementary Fig. 16. RF of example non-overlapping single units.
The color illustrates the magnitude of the units responses to the black rectangle during the RF mapping session. Firing rates of lover than 3 SD are replaced by zero for better illustrations. The white concentric circles used in NCS stimuli are shown for comparison with the RF location of units.

Mouse ID	strain	Gender	Recording day	Recording depth from the surface (μm)
1	PV-Cre	Male	1	NA*
			2	NA
			3	NA
			4	950
			5	900
			6	950
2	PV-Cre	Male	1	NA
			2	NA
			3	NA
3	PV-Cre	Female	1	900
			2	950
			3	800
			4	900
			5	850
4	PV-Cre	Female	1	800
			2	800
			3	850
			4	950
			5	950
			6	1100
			7	1050
			8	850
			9	1050
			10	950
			11	950
5	C57BL/6N	Male	1	850
			2	850
			3	775
			4	800
			5	800
6	C57BL/6N	Male	1	800
			2	850

Supplementary Table 1. Specification of used mice in the V1 recording experiment with the perpendicular angle of electrode penetration. *The exact depth was not recorded.

Mouse ID	strain	Gender	# Recording days
7	PV-Cre	Male	10 days
8	PV-Cre	Female	5 days
9	C57BL/6N	Male	4 days
10	C57BL/6N	Male	10 days
11	C57BL/6N	Male	6 days
12	PV-Cre	Female	4 days
13	PV-Cre	Female	5 days

Supplementary Table 2. Specification of used mice in the V1 recording experiment with an oblique angle of electrode penetration.

Mouse ID	strain	Gender	# Recording days
14	PV-Cre	Male	6 days
15	PV-Cre	Male	3 days
16	PV-Cre	Male	4 days
17	PV-Cre	Female	3 days
18	PV-Cre	Female	4 days
19	PV-Cre	Female	5 days

Supplementary Table 3. Specification of used mice in the optogenetics experiment with oblique angle of electrode penetration.
