# Supplementary material for: Brightness illusions drive a neuronal response in the primary visual cortex under top-down modulation
Source: Nat Commun. 2024 Apr 23;15:3141. doi: 10.1038/s41467-024-46885-6 (PMC11039481; doi:10.1038/s41467-024-46885-6)
Supplement: Supplementary file 3 — Description of Additional Supplementary Files [file 41467_2024_46885_MOESM3_ESM.pdf]

Description of Additional Supplementary Files

File name: Supplementary Movie 1

Description: Neon-Color-Spreading (NCS). Movie demonstration of Neon-Color-Spreading stimulus.

File name: Supplementary Movie 2

Description: Luminance Defined Grating (LDG). Movie demonstration of Luminance-Defined-Grating stimulus.

File name: Supplementary Movie 3

Description: Diffusion-Blocked Control (DBC). Movie demonstration of Diffusion-Blocked Control stimulus.
