# Supplementary material for: Brightness illusions drive a neuronal response in the primary visual cortex under top-down modulation
Source: Nat Commun. 2024 Apr 23;15:3141. doi: 10.1038/s41467-024-46885-6 (PMC11039481; doi:10.1038/s41467-024-46885-6)
Supplement: Supplementary file 7 — Reporting Summary [file 41467_2024_46885_MOESM7_ESM.pdf]

nature portfolio

Corresponding author(s): Masataka Watanabe
Last updated by author(s): Nov 27, 2023

Reporting Summary

Nature Portfolio wishes to improve the reproducibility of the work that we publish. This form provides structure for consistency and transparency in reporting. For further information on Nature Portfolio policies, see our Editorial Policies and the Editorial Policy Checklist.

Statistics

For all statistical analyses, confirm that the following items are present in the figure legend, table legend, main text, or Methods section.

n/a	Confirmed	
☐	☒	The exact sample size (*n*) for each experimental group/condition, given as a discrete number and unit of measurement
☐	☒	A statement on whether measurements were taken from distinct samples or whether the same sample was measured repeatedly
☐	☒	The statistical test(s) used AND whether they are one- or two-sided<br>*Only common tests should be described solely by name; describe more complex techniques in the Methods section.*
☒	☐	A description of all covariates tested
☐	☒	A description of any assumptions or corrections, such as tests of normality and adjustment for multiple comparisons
☐	☒	A full description of the statistical parameters including central tendency (e.g. means) or other basic estimates (e.g. regression coefficient) AND variation (e.g. standard deviation) or associated estimates of uncertainty (e.g. confidence intervals)
☐	☒	For null hypothesis testing, the test statistic (e.g. *F*, *t*, *r*) with confidence intervals, effect sizes, degrees of freedom and *P* value noted<br>*Give P values as exact values whenever suitable.*
☒	☐	For Bayesian analysis, information on the choice of priors and Markov chain Monte Carlo settings
☐	☒	For hierarchical and complex designs, identification of the appropriate level for tests and full reporting of outcomes
☒	☐	Estimates of effect sizes (e.g. Cohen's *d*, Pearson's *r*), indicating how they were calculated

Our web collection on statistics for biologists contains articles on many of the points above.

Software and code

Policy information about availability of computer code

Data collection	Data acquisition was made by Open Ephys GUI (v0.4.4 or v0.4.6) or Cerebus Central Suite (v7.0.4; Blackrock Microsystems LLC). Visual stimuli were programmed and generated in MATLAB (v.R2018a-R2020a, MathWorks, Inc.) and Psychophysics Toolbox Version 3 (PTB-3).
Data analysis	NPMK (V5.0.0.0; BlackrockNeurotech) was used to read recorded data file. We used Kilosort 2 (running on Matlab R2018a)for spike sorting and template-GUI (phy v2.0) for manual curation of sorted clusters. All other analyses were performed using built-in function and custom codes written in Matlab R2020a. CircStat Toolbox was used for circular data analysis. Adobe Illustrator (v 2020) was used to make panel figures.

For manuscripts utilizing custom algorithms or software that are central to the research but not yet described in published literature, software must be made available to editors and reviewers. We strongly encourage code deposition in a community repository (e.g. GitHub). See the Nature Portfolio guidelines for submitting code & software for further information.

Data

Policy information about availability of data

All manuscripts must include a data availability statement. This statement should provide the following information, where applicable:

- Accession codes, unique identifiers, or web links for publicly available datasets
- A description of any restrictions on data availability
- For clinical datasets or third party data, please ensure that the statement adheres to our policy

Spike data have been deposited on Figshare at: https://doi.org/10.6084/m9.figshare.24635439.v1

Research involving human participants, their data, or biological material

Policy information about studies with human participants or human data. See also policy information about sex, gender (identity/presentation), and sexual orientation and race, ethnicity and racism.

Reporting on sex and gender	N/A
Reporting on race, ethnicity, or other socially relevant groupings	N/A
Population characteristics	N/A
Recruitment	N/A
Ethics oversight	N/A

Note that full information on the approval of the study protocol must also be provided in the manuscript.

Field-specific reporting

Please select the one below that is the best fit for your research. If you are not sure, read the appropriate sections before making your selection.

☒ Life sciences ☐ Behavioural & social sciences ☐ Ecological, evolutionary & environmental sciences

For a reference copy of the document with all sections, see nature.com/documents/nr-reporting-summary-flat.pdf

Life sciences study design

All studies must disclose on these points even when the disclosure is negative.

Sample size	No statistical methods were used to pre-determine the sample size. However, sample size (n = 19 mice) was chosen based on standards for replicability of neuronal recording (Y Senzai, A Fernandez-Ruiz, G Buzsáki, Neuron, 2019)
Data exclusions	In six mice, we recorded 636 V1 single units and analyzed the stimulus-evoked spiking of 520 single units. The analysis of response latency was performed only on complex cells (n=340), as the estimation of response latency is not feasible in simple cells. Phase shift analysis was applied only on F1-dominant cells (n=209), which exhibit a phasic response to moving grating. In another sets of experiment we recorded 1807 V1 neurons from 13 mice and used different sub-populations for different analysis as follow: replicating the the main findings with non overlapping RF cells(n=234), latency comparisons (n=236 cells), and opto-inhibition experiments (n=85 cells).
Replication	All experiments were replicated in multiple mice. 6 mice were used to record only V1 response. 7 mice were used to measure the neuronal responses in V1 neurons to full-screen stimuli. Optogenetic experiments were performed using 6 mice.
Randomization	All the visual stimuli as well as light and no light trials (for optogrnrtic experiments) were presented to mice in a pseudo-randomized order.
Blinding	Blinding was not possible in this study because there were no experimental groups. But the surgical procedure, data acquisition, and curation were done by 2-3 lab members.

Reporting for specific materials, systems and methods

We require information from authors about some types of materials, experimental systems and methods used in many studies. Here, indicate whether each material, system or method listed is relevant to your study. If you are not sure if a list item applies to your research, read the appropriate section before selecting a response.

Materials & experimental systems

n/a	Involved in the study
☒	☐ Antibodies
☒	☐ Eukaryotic cell lines
☒	☐ Palaeontology and archaeology
☐	☒ Animals and other organisms
☒	☐ Clinical data
☒	☐ Dual use research of concern
☒	☐ Plants

Methods

n/a	Involved in the study
☒	☐ ChIP-seq
☒	☐ Flow cytometry
☒	☐ MRI-based neuroimaging

Animals and other research organisms

Policy information about studies involving animals; ARRIVE guidelines recommended for reporting animal research, and Sex and Gender in Research

Laboratory animals	Data acquisition was done using 19 adult C57BL/6 or PV-Cre mice (homozygous for the PV-Cre genes, B6;129P2-Pvalbtm1(cre)Arbr/J) with minimum age of 8 weeks.
Wild animals	This study did not involve wild animals.
Reporting on sex	This study did not include any sex-based analyses.
Field-collected samples	No field-collected samples were used.
Ethics oversight	The local authorities (Regierungspräsidium Tübingen) approved all animal procedures and the procedures were done in compliance with EU Directive 2010/63/EU (European Community Guidelines for the Care and Use of Laboratory Animals).

Note that full information on the approval of the study protocol must also be provided in the manuscript.
